# Oxytocin predicts positive affect gains in a role-play interaction

**DOI:** 10.3389/fpsyg.2024.1258254

**Published:** 2024-05-30

**Authors:** Alexandru I. Berceanu, Claudiu Papasteri, Alexandra Sofonea, Romina Boldasu, Diana Nita, Cătălina Poalelungi, Robert Froemke, Ioana Carcea

**Affiliations:** ^1^Cognitive Development and Applied Psychology through Immersive Experiences, LDCAPEI, CINETic Centre, National University of Theatre and Film I. L. Caragiale, Bucharest, Romania; ^2^Department for Animation and Interactivity, National University of Theatre and Film I. L. Caragiale, Bucharest, Romania; ^3^Department of Psychology, Faculty of Psychology and Educational Sciences, University of Bucharest, Bucharest, Romania; ^4^Acting Department Theatre Faculty, National University of Theatre and Film I. L. Caragiale, Bucharest, Romania; ^5^National Institute of Endocrinology C. I. Parhon, Bucharest, Romania; ^6^Skirball Institute for Biomolecular, School of Medicine, New York University, NY, United States; ^7^Department of Pharmacology, Physiology and Neuroscience, New Jersey Medical School, Rutgers, Brain Health Institute, The State University of New Jersey, Newark, NJ, United States

**Keywords:** role-play, oxytocin, positive affect, prosocial attitudes, emotion regulation

## Abstract

**Introduction:**

Role-play, a key creative process in theatre, is used in therapeutic interventions to improve social skills, emotion regulation, and memory. Although role-play is widely used as a psychotherapeutic technique, its mechanisms of action are not fully understood.

**Methods:**

Our study introduces a standardized controlled procedure for promoting role-play in the laboratory based on the portrayal of a fictional persona and examines its effects on anxiety, affect, prosocial attitudes, and salivary oxytocin dynamics in 38 participants.

**Results:**

In our experiment, role-play significantly increased positive affect and prosocial attitudes and decreased anxiety compared to a control condition. Basal salivary oxytocin levels predicted higher gains in positive affect following role-play, suggesting a specific moderating effect of oxytocin. The fictional persona used in the procedure was rated as very happy by subjects, creating a positive social context for the role-play social interaction.

**Discussions:**

We propose that the observed moderation effect of oxytocin in our study is specific to the role-play condition due to the capacity of role-play to generate an affective regulatory context based on congruency toward the emotional state of the fictional persona. Our findings indicate that basal oxytocin levels could predict specific outcomes of role-play in therapeutical setting. We discuss several psychological and biological mechanisms that could account for the observed effects of role-play and how oxytocin could act as a substrate for them.

## Introduction

The neurohormone oxytocin received a lot of attention for its involvement in social behaviors, especially in maternal behavior (Pedersen et al., 1982; Marlin et al., 2015; Carcea et al., 2021), reproductive behavior (Insel et al., 1997) but also for its role in affiliative behaviors between partners (Williams et al., 1994; Insel and Hulihan, 1995), prosocial behavior and social cognition in humans. Currently, it is proposed that oxytocin’s role in social behaviors emerges from oxytocinergic modulation of the salience of social and emotional stimuli (Insel and Young, 2001; Resendez et al., 2020; Alaerts et al., 2021; Smith et al., 2021; Biggs and Hammock, 2022).

Oxytocin has a complex and multifaceted role in social behavior, and has been linked to both positive and negative social interactions (Papasteri et al., 2020), prosocial behaviors, but also aggression or envy (Shamay-Tsoory and Abu-Akel, 2016; Poore and Waldman, 2020). The impact of oxytocin at the behavioral level is dependent on gender (Ma et al., 2018), experience (Londono Tobon et al., 2018; Riem et al., 2020), and context (Olff et al., 2013; Hurlemann and Scheele, 2016; Baettig et al., 2020; Handlin et al., 2023).

Play can be seen as an intriguing behavior, with no direct or material benefit for the subject engaging in play (Caillois, 2001). The proposed evolutionary purpose of play is to facilitate exercising skills, behavioral acquisition, and context adaptation, with proposed health and stress-related benefits (Panksepp, 1993; Burghardt, 2005; Schank, 2015). Play behavior is proposed to have important implications in neurodevelopment and neural proliferation (Panksepp, 1993). The neurohormonal substrate for social play behavior is complex with oxytocin observed to modulate social play in animal models dependent on sex and social context (Bredewold et al., 2014; Reppucci et al., 2018). It is proposed that play occurs only in positive emotional states favoring the acquisition of new skills and behaviors (Panksepp, 2000; Olčar, 2013). Positive emotions promote learning, experimenting and creativity (Fredrickson, 2001; Alexander et al., 2021). Oxytocin is strongly related to positive affect (Uvnäs-Moberg, 1998; Brockington et al., 2021). However, it remains unclear if oxytocin acts directly to increase positive affect, or indirectly, as a consequence of its proposed anxiolytic effect (Alexander et al., 2021).

While playing animals and humans generally perform behaviors similar to the ones necessary for survival (hunting, fighting, hiding) repetitively without actually fulfilling the survival objective of the behavior (for instance feeding or mating). Theatrical performance can be conceptualized as a form of play that relies on simulation and mimicry, drawing its complexity on childhood pretense and imitation play (Caillois, 2001). Role-play, a specific form of pretend play, is fundamental to theatrical performance, contributing to the creation of the fictional reality in the theatrical setting. Building on the suggested objectives of play and its relation to positive affective states, we propose the use of role-play to promote positive affect and prosocial attitudes and to modulate salivary levels of oxytocin.

It is considered that role-play relies on the human capacity of theory of mind, the process of inferring other people’s intentions, thoughts, and emotions (Goldstein, 2018; Mirodan, 2018; Brown et al., 2019). To create roles and immerse themselves in “the fictional character behavior,” children, adults, and professional actors engage in complex cognitive processes such as imagination, memory, theory of mind, executive control, verbal skills, emotion regulation, and others (Goldstein, 2009). Through role-play, individuals partake in simulated actions by adhering to the “as-if” principles that govern the generation of coherent fictional behaviors modeled from reality (Yardley-Matwiejczuk, 1997; Stanislavski, 2010).

Role-play is widely used in psychotherapy, assessment, and psychology research (Yardley-Matwiejczuk, 1997; Landy et al., 2003). Role-play is used in creative and child-oriented therapies (Levenson and Jack, 1991; Folostina et al., 2015; Havens, 2019) cognitive behavioral therapy (Butler et al., 2006; Dagnan and Jahoda, 2006; Morgan and Banerjee, 2006; Clarck and Beck, 2010; Dagnan et al., 2018), interpersonal therapy, (Weissman et al., 2000; Stangier et al., 2011), and many others (Sterling, 1993; Corsini, 2010; Wedding and Corsini, 2013). The use of role-play in all these approaches assumes that through role-play one could obtain a new image of oneself and of the relationship with the surrounding world through enactive learning, exercising new behaviors and gaining emotional control (Corsini, 2010; Goldstein, 2018).

Social psychologist Goffman (1959) proposed that people adopt roles in their lives based on the social context to maximize their opportunities. Intricate “ifs” and “as ifs” construct a social image of the self, dependent on social context. Adopting stereotypical roles in different social contexts (for instance, adopting the role of “the father” in a work context) is determined by a lack of flexibility, an aspect considered determinantal for psychological well-being and social functioning (Moreno, 1947; Landy, 2009). It is proposed that spontaneity can be trained through role-play (Landy, 2009). Drama therapy proposes role-play as beneficial to the client’s psychological health through the diversification of the repertoire of performed behaviors and social roles according to context (Moreno, 1946, 1947; Emunah, 2009; Landy, 2009).

It is widely considered that individuals involved in role-play “merge” at some level during a performance with the fictional persona by both professionals and the public (Goldstein, 2018). Anectodical stories, plays and films talk about actors who were not able to get out of their role in the fictional world after the performance ended such as presented in the film Farewell My Concubine, the first Chinese film to receive Palm D’Or at Cannes (Kaige, 1993). Here, the main character takes the role of a concubine from the opera he was playing and exerts it in his real life. To create a realistic portrayal of a character some actors purposefully live in conditions like the ones experienced by the fictional persona. For example, the actor Adrien Brody gave up his apartment, sold his car, and moved to Europe experimenting with a scarce life, like the one of the characters he was to portray in The Pianist (Polanski, 2002) for which he received the Oscar for Best Actor.

Children develop the capacity to quarantine imagined worlds from invading their reality as early as they engage in pretend play (Goldstein and Bloom, 2015; Goldstein, 2018). In the context of acting, “merger” is sometimes called “carryover” and was documented both for actors involved in film (Brown, 2019) and in theatre (Berceanu et al., 2020; Hatami, 2023). It is described by actors as carrying psychological states from performance to personal life, both positive and negative emotions, as well as attitudes or behaviors (Brown, 2019; Hatami, 2023). It is supposed that professional actors use distancing to quarantine reality from pretend reality (Goldstein, 2018; Mirodan, 2018) Although often adults prefer realistic portrayals of characters (Goldstein and Bloom, 2015) “merger” is not synonymous with a good character portrayal or high-quality acting. The “merger” aspect of role-playing is not thoroughly studied (Goldstein, 2018), and its clarification would be beneficial not only to professional persons engaging in role-playing but to all categories using role-play, especially the ones using it in clinical settings.

Our purpose was to develop a standardized procedure for inducing positive affect and prosocial attitudes through role-play for participants with no prior training in acting. Our research question is if role-playing a positive fictional persona could promote prosocial attitudes, positive emotions, and reduce anxiety and negative affect and if this changes could me mediated by oxytocine. Answering this question could encourage the potential use of role-play as an intervention in clinical settings.

Our hypothesis is that role-play increases positive affect and prosocial attitudes in congruency with the fictional persona, and that these changes correlate with salivary oxytocin levels. Our results could enhance the use of role-play in psychotherapy by clarifying the role of “merger” as an underlying mechanism contributing to the observed benefits in various forms of psychotherapeutic approaches employing role-play.

## Methods and design

The use of role-play in research was widely critiqued for lack of consistency in induction methods (Yardley-Matwiejczuk, 1997). Role-play impact on affect level was previously shown to be dependent on context, content, and performance style (Kipper and Har-Even, 1984; Herrera et al., 2018; Berceanu et al., 2020). To ensure a controlled experimental context, we developed a standardized procedure for role-play induction. Variations of this method are used both with children, adults as well as in acting programs and it is inspired by Viola Spolin’s exercise, “The Radio” (Spolin et al., 1999). The exercise consists of an interviewee answering questions posed by an interviewer. The interviewee answers the questions from the perspective of a fictional persona (or real character). The interviewee uses all available character attributes as “as-if” instructions during the process. In some more complex requirements, the interviewee could also use specific gestures or costumes to generate an embodied representation. Role-play induction by answering questions from the perspective of a character was previously used in research (Brown et al., 2019).

### Participants

Forty-two adults with ages between 19 and 64 (*M* = 39.9, SD = 10.1) enrolled in a psychology program volunteered to participate in the study, 34 were women (81%). The participants did not have specific theatre training. Three days were required for full participation in the study. Some of the participants did not fulfill the third day of the study, therefore missing one of the experimental conditions, either “Self” or “Role-play.” Four participants completed the “Role-play” condition (but not the “Self” condition), and some other four participants completed the “Self” condition (but not the “Role-play” condition). Thirty-seven participants completed the “Role-play” condition and 36 completed the “Self” condition bringing a total of 8 drop-out for the third day of the experiment. If a subject participated in one condition, we have included the data in the analysis. No inclusion or exclusion criteria were used except checking for theatre training experience, none of the enrolled participants stated to have prior training in acting.

Drop-out was determined by the impossibility of scheduling the third participation in the study in a reasonable time frame (one month after the first day) due to the schedule of the subject. Including more subjects in the experiment to obtain the target number of participants per condition was impossible as the emergency state was declared and no more in-lab data collection was possible for several months due to the COVID-19 global pandemic. One subject participated in the study in the “Self” condition but due to an error in record tracking post responses were lost and all his data were excluded from the analysis, consequently, data from 41 subjects was included in our analysis.

### Ethics

The study received approval from the University’s Ethics Committee. Participants were recruited through announcements to students enrolled in a psychology program. Voluntary participants were rewarded with research practice hours credit as an incentive for their participation. Participants were informed about the study and were enrolled after expressing their agreement and signing the informed consent form.

### Design

Our study employed a randomized crossover design. The experiment consisted of a three-day protocol: day one—training, day two—dramatic action in either the “Role-play” or “Self” condition and day three—“dramatic action” in the remaining condition. On the first day, assigned for training, participants performed both the “Self” condition and the “Role-play” on a short set of 12 interview questions.

**Figure 1:**
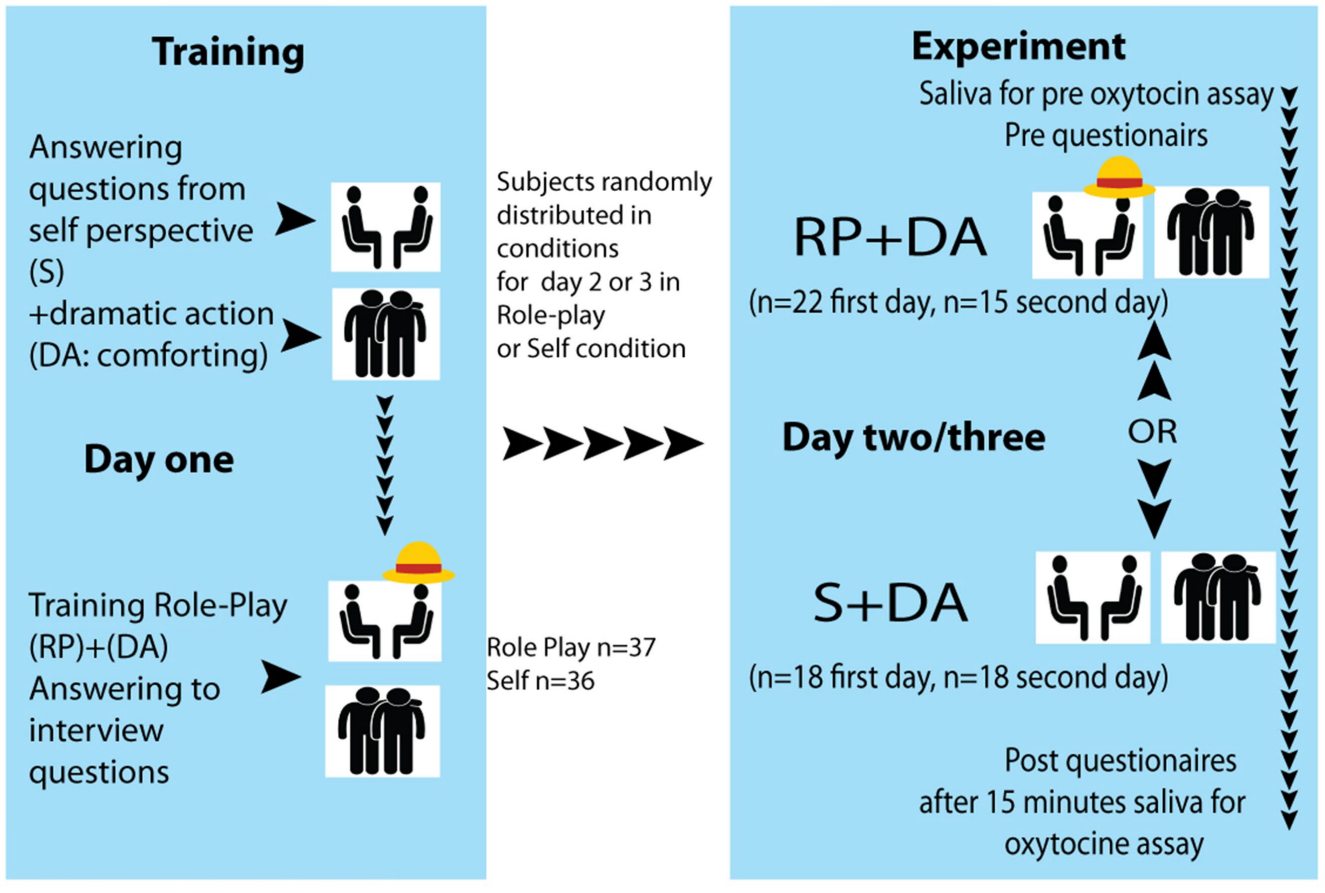
Experimental design.

Training and questionnaires were applied by an experimenter. Interviews were conducted by a different person, the “interviewer.” The experiment was conducted by three different women experimenters and three different women interviewers. The second and third days of the study consisted of the “Role-play” or “Self” conditions. Ordering was balanced, despite the dropouts. In the final sample, 18 of the participants were allocated to participate in the “Self,” and 22 in the “Role-play” condition on the second day, and 18 subjects in the “Self” and 15 in the “Role-play” on the third day of participation in the study (see Figure 1 experimental design).

### Role-play induction script

To ensure a standardized role-play induction method for our study, a specially developed scripted account of the life of a fictional character was audio-recorded along with all other instructions (see in Supplementary material 1). The script followed the story of an “unlucky” person, focusing on several funny, but also challenging events. The story depicted the character as someone who encountered continuous misfortunes but remained optimistic and prosocial. For the fictional persona adversities would ultimately transform into positive outcomes, resulting in valuable experiences overall.

A professional actress was audio-recorded narrating the script as the experience of a friend in the third person. The story was recorded in both “she” and “him” versions to align with the gender of the participant listening to them. The duration of the recording was 4 min and 45 s for the “him” version and 4 min and 35 s for the “her” version, with no other changes in the script besides gender pronouns.

### Experimental conditions

In our study subjects participated in two conditions: “Self” and “Role-play.” In both conditions, subjects were answering to the same set of questions asked by the interviewer. In the “Self” condition subjects were instructed to answer the question from their perspective with the intent to comfort the interviewer. In the “Role-play” condition subjects were asked to answer questions from the perspective of the fictional persona with the same intent of comforting the interviewer.

**Figure 2:**
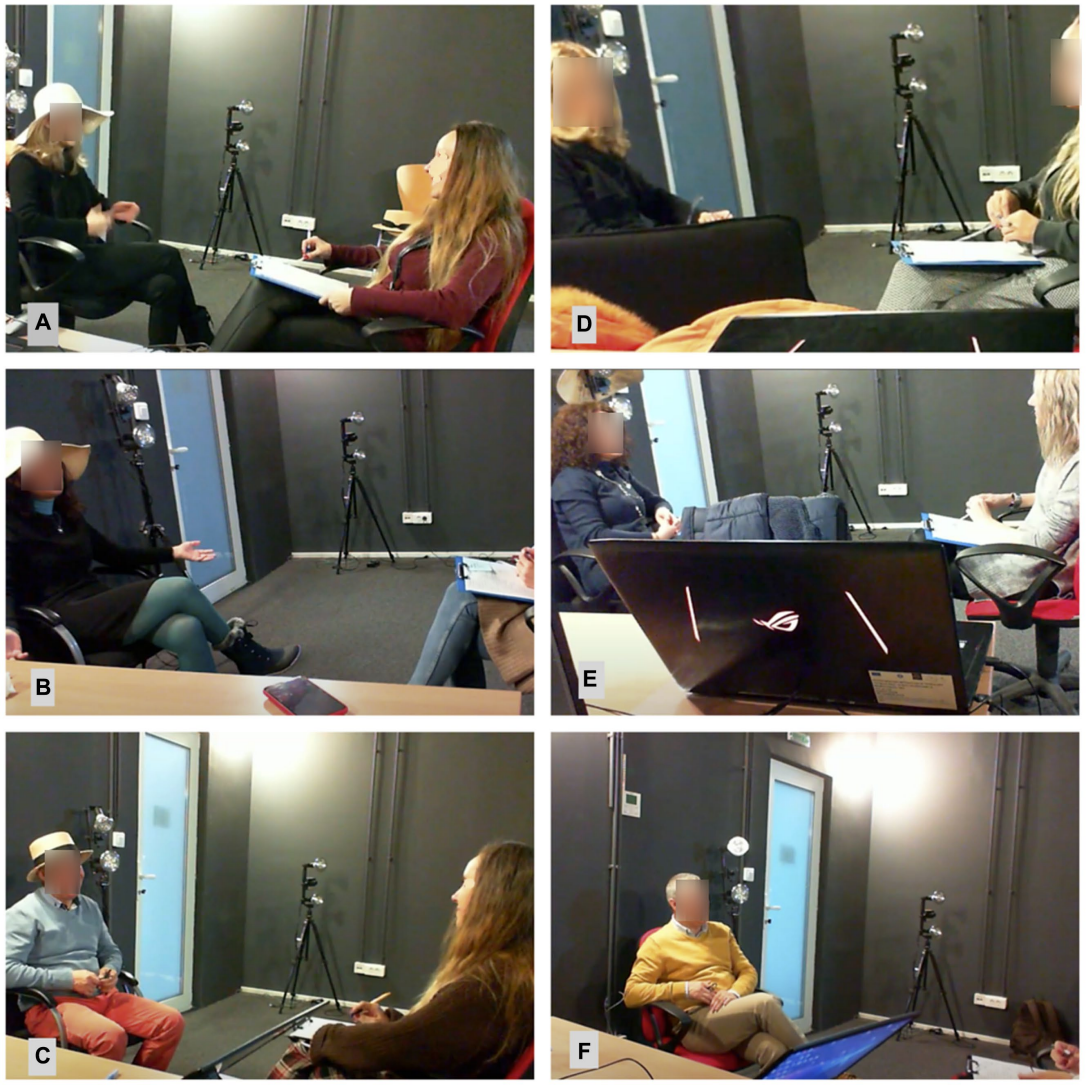
Subjects in the “Role-play” condition **A–C** and in the “Self condition” **D–F**.

In the role-play condition subjects were asked to wear a hat they chose as specific to the fictional persona (Figure 2). Using objects specific to the character is generally considered in acting training as an important help to keep the perspective of the character. The role-play instruction would state: “I will describe a character to you. At the end of the story, you will put on his hat, which is on the table, and you will answer if you were him/her. In the end, you will take off the hat and become yourself again. While wearing the hat, try to view the world and everything that happens from his perspective and act and answer as he/she would to all questions. He/she will have your voice and your body; there is no need to make any extra effort other than wearing his hat.”

### Day one—training

During the training session, participants were requested to answer a series of six preselected training questions such as “What is your favorite pet?” first from their perspective (the complete list is provided in the Supplementary material 1). Following the playback of the recorded narration, participants were instructed to assign a suitable name to the character, avoiding their name or that of a close friend or family member. After listening to the story subjects were instructed to rate their perceptions of the character using a Visual Analog Scale (VAS) on the Character Evaluation Scale (CES) indicating the levels of perceived positivity, helpfulness, and prosocial behavior of the character. After listening to the story, subjects were also asked to give a name to the character. In the role-play condition, the experimenter would address the questions to the participant using the name assigned by each of them to the character. If a participant would name the character “Star” a question would be addressed in the role-play condition “Star, what is your favorite pet?.” In the recorded story the Unlucky had a dog pet so answering from the perspective could be based on one of the elements about the dog in the story. While training if the participant’s answers significantly deviated from the character’s perspective, as in answering with the same pet they provided as their preference, the experimenter would exemplify the difference of perspective to guide them in the role-play condition. For instance, when asked what to do if they forgot their keys at home, a role-play example also including a dramatic action response would be: “No worries! I once lost my keys, but this led to new connections and positive experiences, I have met a locksmith. Focus on your surgery and hope for the best, I could go find them for you, but I do not think it’s such a good idea, most probably it could start a small disaster.” A response from self-perspective, including the dramatic action of comforting would be: “Wait here I will go get them before your surgery ends.” In both examples, the dramatic action of comforting is included but in role-play answer, we have also according to the fictional persona way of seeing the world the unfortunate event of losing keys as a positive o event since one will need to meet to locksmith to break the door. In both situations, the subject is pretending that the interviewer will have surgery and needs comforting, but only in the role-play perspective he/she sees all events as turning well.

Some of the questions had answers directly relating to the narration (for instance “What do people say about you?,” the story depicting the perspective of others as liked, funny, warm, and positive), while for other questions no direct information would be available (for instance, “You hear a kitten crying in the dark, what do you do?”). Participants were instructed to provide detailed answers in both conditions and to try to attain the “dramatic action” objective of comforting the interviewer before their surgery.

### Days two and three—“Role-play” intervention or control: “Self”

After finishing the training session, participants would be randomly assigned to perform the “Self” or the “Role-play” condition on the second day and the remaining condition on the third, the days being non-consecutive. The average number of days between conditions was *M* = 11.14 with SD = 9.26.

The experimenter assisted the participant in completing questionnaires before and after social interaction. A saliva sample was collected 15 min after the participant arrived at the lab. The instructions for the assigned condition were played, followed by the interviewer entering the room. The interviewer posed the same set of 30 questions and then exited the room. Next, the experimenter returned to the room and asked the participant to complete the post questionnaires, followed by another saliva sample collected after 15 min of “Self” or “Role-play” interaction ended.

### Instructions and interview questions

For each condition, the instructions for the task were played through a loudspeaker located in the experimental room with the subject being alone in the room. The recordings conveyed the main guidelines explained in training. For the control condition, participants were instructed as follows: “Answer the questions from your own perspective.” In the “Role-play” condition, participants were prompted as previously stated in its description. Finally, the instruction for the “dramatic action” was played in both conditions, stating: “The person entering the room is scheduled for a surgical procedure shortly after the interview, comfort her with your answers.” The interviewers would solely ask the questions without displaying any behavioral signs that might relate to a future surgery. All participants were video-monitored and adherence to protocol was assessed by experimenters.

### Dramatic action

To distinguish role-play effects from other types of pretend-play effects, our subjects performed a control pretend-play task, present both in the “Role-play” and control condition, in the form of a “dramatic action” instruction: “The person that is about to enter the room will very soon go through a scheduled surgical procedure. Try to comfort her with your answers.” In this context, “dramatic action” is defined as the intentional effort made to influence or alter the state of another individual. It serves as a practical framework for establishing a goal within a theatrical performance (Liron et al., 2018). “Dramatic actions” are based on unequivocal active verbs which are concrete, but also have a sense of generality, like: “soothe,” “comfort,” “hurt,” “encourage” or “cheer,” and are used as director instruction or improvisation prompts. High inter-participant agreement on dramatic-action verbs, their graphical representation, meaning, and emotional valence (Liron et al., 2018) recommended the use of “dramatic actions” within a controlled design.

### Instruments

#### Oxytocin assay

Saliva samples were collected two times for OXT assay as previously reported through Radio-Imuno-Assay, RIA, (Landgraf et al., 1982; Papasteri et al., 2020; Tomescu et al., 2022). Participants provided saliva samples after 15 min after arriving in the lab without social contact and 15 min after the experimental and control conditions, assisted by a qualified medical nurse. The saliva samples were collected in special recipients (Salivette, Sarstedt). The participants were instructed to move the synthetic swab found inside the salivette slowly in their mouth until it was saturated with saliva. The swab was then placed back into the specific recipient and centrifuged at 1.000 g, at 4°C for 2 min and the samples were aliquoted in 1.5 mL Eppendorf vials that were stored at −80°C prior to analysis. OXT was measured by radioimmunoassay (RIA) at RIAgnosis, Munich, Germany, while total proteins were measured at the National Institute of Endocrinology “C. I. Parhon,” Bucharest, Romania. Salivary total protein was used to normalize the concentration of salivary OXT levels since its concentration can vary significantly with saliva viscosity.

##### Adult Prosocialness Scale

Adult Prosocialness Scale (APS) is a 16-item questionnaire with responses on a five-points Likert scale developed to assess the global propensity to behave prosocial from late adolescence to adulthood (Caprara et al., 2005). The Adult Prosocialness Scale was designed as a measure to assess individual differences in general adults’ tendencies to act in favor of others and has been proven useful in several studies in different countries (Caprara et al., 2005). The scale has been validated in Italy with classical test theory and the item response theory approach showing adequate psychometric qualities and construct validity (Kanacri et al., 2021). APS has been correlated with agreeableness and emotional and empathic self-efficacy (Alessandri et al., 2009). To observe if the attitudes of subjects are impacted by role-play, we chose a scale aimed at observing prosocial traits and we decided to apply it before and after our task. Items are represented by statements that reflect prosocial feelings and behaviors, e.g., “I spend time with those friends who feel lonely.” Participants respond on five-point Likert scales, with 1 representing never/rarely true and 5 representing almost always/always true. The psychometric properties of the scale have been investigated in Italy first (Caprara et al., 2005), more recently in a series of Spanish-speaking countries (Martí-Vilar et al., 2020), followed by a cross-national investigation, comparing data from different Western and non-western cultures (Kanacri et al., 2021), all showing adequate psychometric qualities and construct validity with a Cronbach’s *α* for the entire set of items of 0.94 (Caprara et al., 2005).

##### The State-Trait Anxiety Inventory

The State-Trait Anxiety Inventory (STAI-Y) is a commonly used measure of trait and state anxiety used in clinical settings to diagnose anxiety and to distinguish it from depressive syndromes (Spielberger, 1989). It has 20 items for assessing trait anxiety and 20 for state anxiety (Spielberger, 1989). State anxiety items include items like: “I am tense; I am worried” and “I feel calm; I feel secure,” higher scores indicate greater anxiety (Spielberger et al., 1983). Internal consistency coefficients are good with a Cronbach’s *α* ranging from 0.86 to 0.95; test-retest reliability coefficients have ranged from 0.65 to 0.75 over a 2-month interval (Spielberger et al., 1983; Spielberger, 1989) Due to later inclusion of the instrument in the study, only 28 participants completed the STAI-Y.

##### The Positive and Negative Affect Schedule

The Positive and Negative Affect Schedule (PANAS) is a scale widely used to measure mood or emotion (Watson et al., 1988). It consists of 20 items, with 10 items measuring positive affect (e.g., excited, inspired) and 10 items measuring negative affect (e.g., upset, afraid). Each item is rated on a five-point Likert Scale, ranging from 1 = Very Slightly or Not at all to 5 = Extremely, to measure the extent to which the affect has been experienced in a specified time frame. The PANAS was designed to measure affect in various contexts such as now, the past day, or interval ranging to year, or in general (on average). The scale can be used to measure state affect, dispositional or trait affect, emotional fluctuations throughout a specific period, or emotional responses (Tran, 2013). In our study we have used the instruction “How you feel now,” with answers reflecting state affect. The questionnaire is reported to have good psychometric properties, with a Cronbach’s alpha of 0.94 reflecting high internal consistency (Watson et al., 1988).

##### Character Evaluation Scale

Character Evaluation Scale (CES) the Visual Analog Scales (VAS) are 10 cm long unmarked visual scales ranging from 0 to 100. The momentary perceptions of stress and well-being were evaluated on two such scales with 0 reflecting no stress/worst well-being and 100 most stressed ever/perfect well-being. Participants completed them before and after each session (Streiner and Norman, 1989). To evaluate the perception of the fictional character, we created a five-item visual analog rating scale (VAS). The scale featured a sentence on the right and another on the left, instructing participants to mark their opinion at what level the character could be placed on the scale. The five items included were: “The character cares about others/does not care about others,” “The character is sad/happy,” “The character does not help others/helps others,” “The character does not do good for others/does good for the others,” “The character is not agreeable/is agreeable.”

## Results

### Character perception

On the CES five-item Visual Analog Scale the character was perceived as caring for the others (83.6 ± 20.5), happy (77.1 ± 22.8) helping the others (84.3 ± 17.1), doing good for others (84.2 ± 18.8) and agreeable (80.8 ± 21.4).

### Character given names

Given names ranged from nicknames (Fun Face, Bubu, and Busy Head) to common names such as Alice, Mia and Nico names such as Star or Beta.

### Effects of role-play on affect

**Figure 3:**
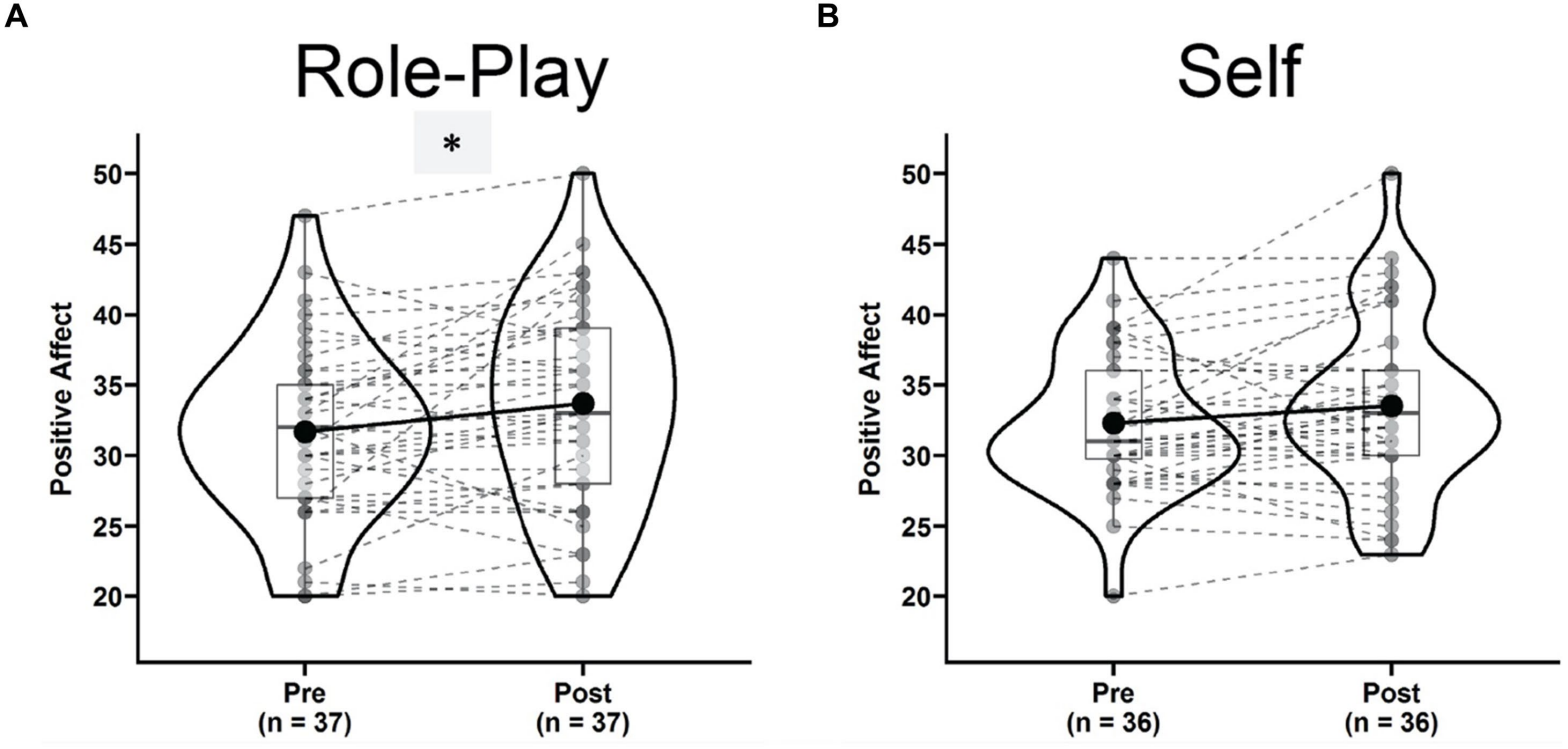
Violin plots of positive affect in the role-play condition **(A)** and self condition **(B)** comparing pre (left) versus post (right) states. ^*^Indicates a significant dynamic.

Positive affect increased significantly after the “Role-play” condition [Figure 3A, Pre: 31.34 ± 6.47, Post: 33.67 ± 7.23, *t*(36) = 2.62, *p* = 0.013, *g* = 0.42, 95% CI = (0.09, 0.76), power_*α* = 0.05_ = 0.704]. After the “Self” condition, participants only had a non-significant marginal increase in positive affect [Figure 3B, Pre: 32.35 ± 4.85, Post: 33.50 ± 6.19, *t*(35) = 1.83, *p* = 0.075, *g* = 0.30, 95% CI = (−0.03, 0.63), power_*α* = 0.05_ = 0.414]. The positive trend in the two conditions was captured by a significant main effect of time in repeated measures ANOVA [*F*(1,31) = 9.474, *p* = 0.004, *η*^2^_g_ = 0.020, power_*α* = 0.05_ = 0.878]. However, due to very low power, the Time × Condition interaction failed to reach significance [*F*(1,31) = 0.037, *p* = 0.848, *η*^2^_g_ = 0.001, power_*α* = 0.05_ = 0.054].

**Figure 4:**
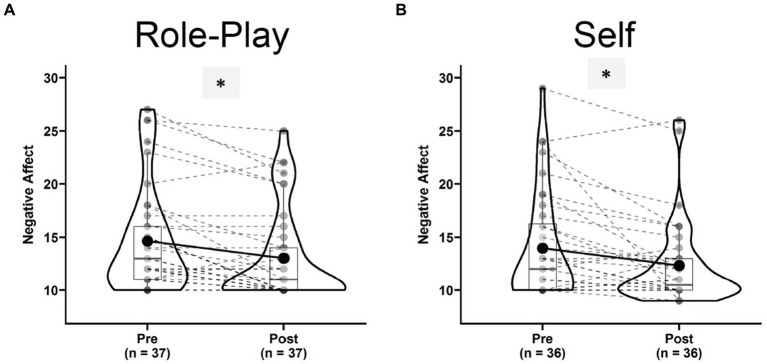
Violin plots of negative affect in the role-play condition **(A)** and self condition **(B)** comparing pre (left) versus post (right) states. ^*^Indicates a significant dynamic.

Negative affect decreased significantly both after the “Role-play” [Figure 4A, Pre: 14.60 ± 4.92, Post: 13.02 ± 4.33, *t*(36) = −0.67, *p* < 0.001, *g* = −0.67, 95% CI = (−1.03, −0.32), power_*α* = 0.05_ = 0.978] and after the “Self” conditions [Figure 4B, Pre: 13.91 ± 5.03, Post: 12.33 ± 3.95, *t*(35) = −3.05, *p* = 0.004, *g* = −0.50, 95% CI = (−0.85, −0.16), power_*α* = 0.05_ = 0.826], with a larger effect sized for “Role-play.” The decreasing trend in the two conditions was revealed by a significant main effect of Time in repeated measures ANOVA [*F*(1,31) = 17.395, *p* < 0.001, *η*^2^_g_ = 0.036, power_*α* = 0.05_ = 0.988]. But again, the Time × Condition interaction failed to reach significance [*F*(1,31) = 0.260, *p* = 0.613, *η*^2^_g_ = 0.001, power_*α* = 0.05_ = 0.081] due to lack of statistical power.

### Effects of role-play on prosocial attitudes and anxiety

**Figure 5:**
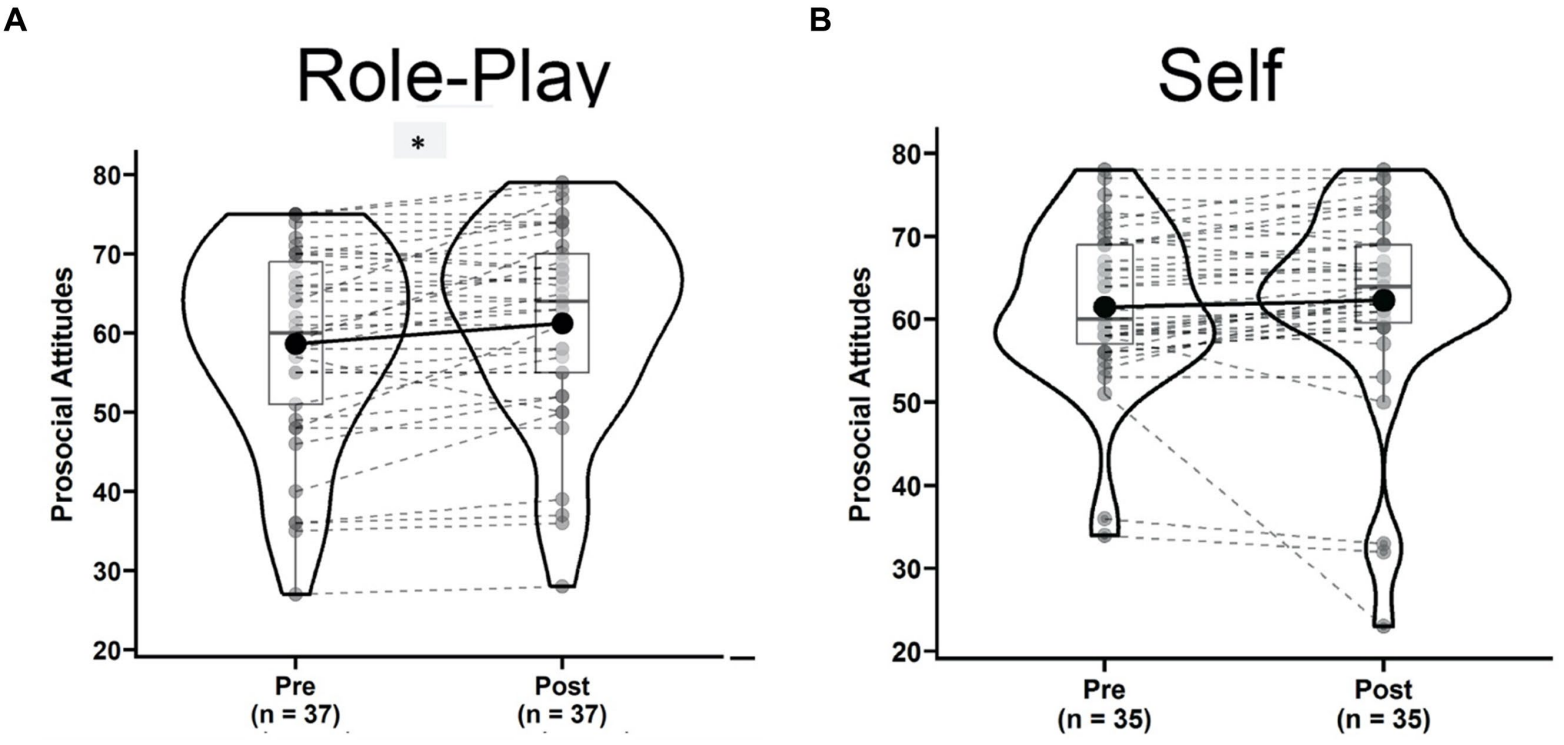
Violin plots of prosocial attitudes in the role-play condition **(A)** and self condition **(B)** comparing pre (left) versus post (right) states. ^*^Indicates a significant dynamic.

The “Role-play” condition was associated with a significant increase in perceived Prosocial Attitudes [Figure 5A, Pre: 58.68 ± 12.39, Post: 61.18 ± 12.36, *t*(36) = 3.42, *p* = 0.002, *g* = 0.54, 95% CI = (0.21, 0.89), power_*α* = 0.05_ = 0.903]. This was not the case for the “Self” condition [Figure 5B, Pre: 61.64 ± 9.53, Post: 62.31 ± 12.27, *t*(34) = 0.75, *p* = 0.458, *g* = 0.12, 95% CI = (−0.21, 0.45), power_*α* = 0.05_ = 0.110]. In the repeated measures ANOVA, the Time × Condition interaction failed to reach significance [*F*(1,31) = 2.382, *p* = 0.133, *η*^2^_g_ = 0.002, power_*α* = 0.05_ = 0.348]. Although, statistical power still lacked, we observed a significant main effect for Time [*F*(1,31) = 4.393, *p* = 0.044, *η*^2^_g_ = 0.005, power_*α* = 0.05_ = 0.567].

**Figure 6:**
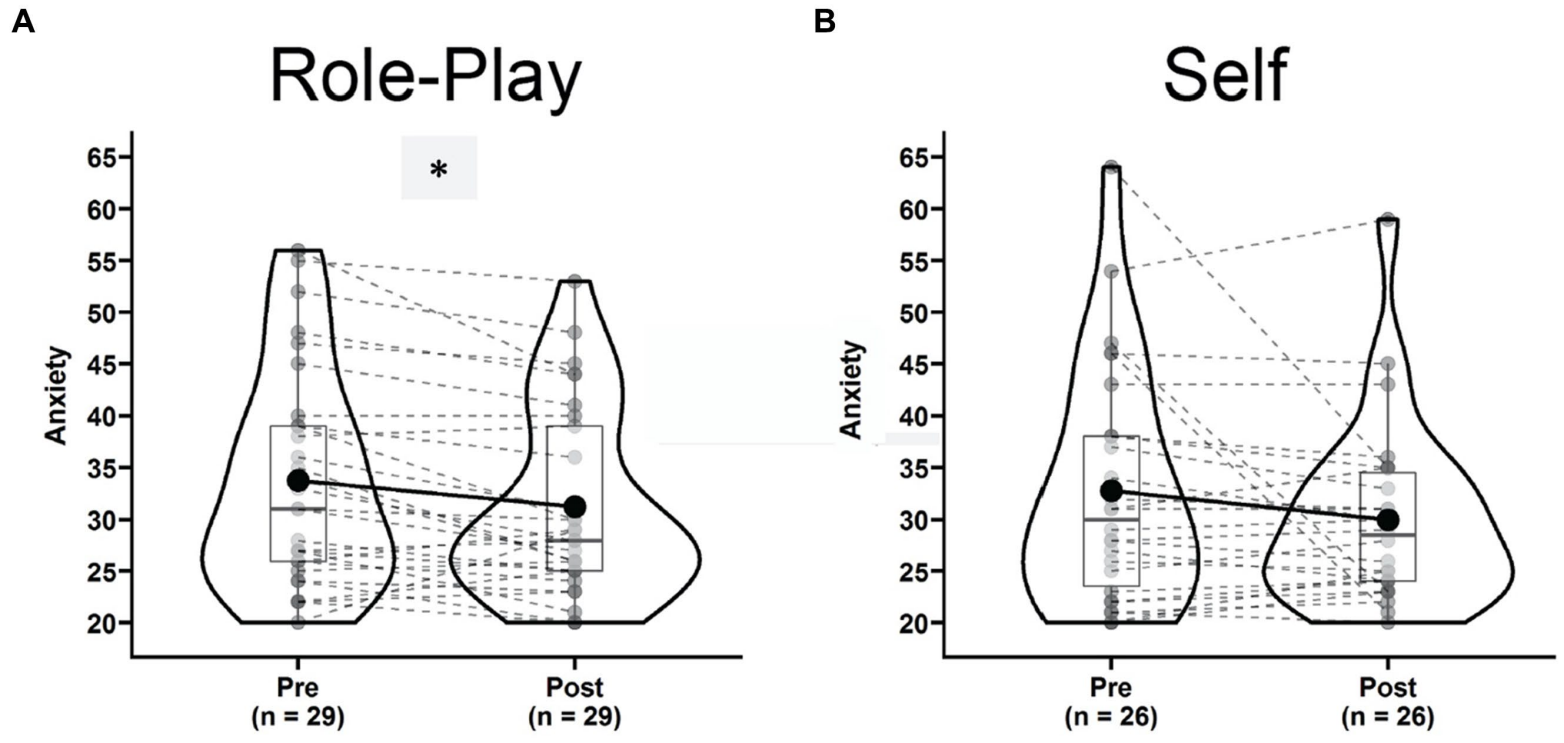
Violin plots of anxiety scores for subjects in the role-play condition **(A)** and self condition **(B)** comparing pre (left) versus post (right) states. ^*^Indicates a significant dynamic.

The anxiety measure was completed only by 29 participants in the “Role-play” condition and 26 in the “Self” condition. Still, anxiety levels where significantly decreased after “Role-play” [Figure 6A, Pre: 34.00 ± 10.54, Post: 31.24 ± 9.10, *t*(28) = 3.22, *p* = 0.003, *g* = 0.58, 95% CI = (0.20, 0.98), power_*α* = 0.05_ = 0.857], but not after “Self” [Figure 6B, Pre: 33.00 ± 10.28, Post: 30.00 ± 8.78, *t*(25) = 1.60, *p* = 0.121, *g* = 0.30, 95% CI = (−0.80, 0.70), power_*α* = 0.05_ = 0.321]. Similar to other results, the decreasing trend within conditions was revealed by a significant main effect of Time in repeated measures ANOVA [*F*(1,19) = 5.653, *p* = 0.028, *η*^2^_g_ = 0.026, power_*α* = 0.05_ = 0.869]. Nevertheless, the sample size available for these analyses was very small and statistical power lacked severely. The Time × Condition interaction failed to reach significance [*F*(1,19) = 0.111, *p* = 0.743, *η*^2^_g_ = 0.001, power_*α* = 0.05_ = 0.071].

### Effects of role-play on salivary oxytocin levels

**Figure 7:**
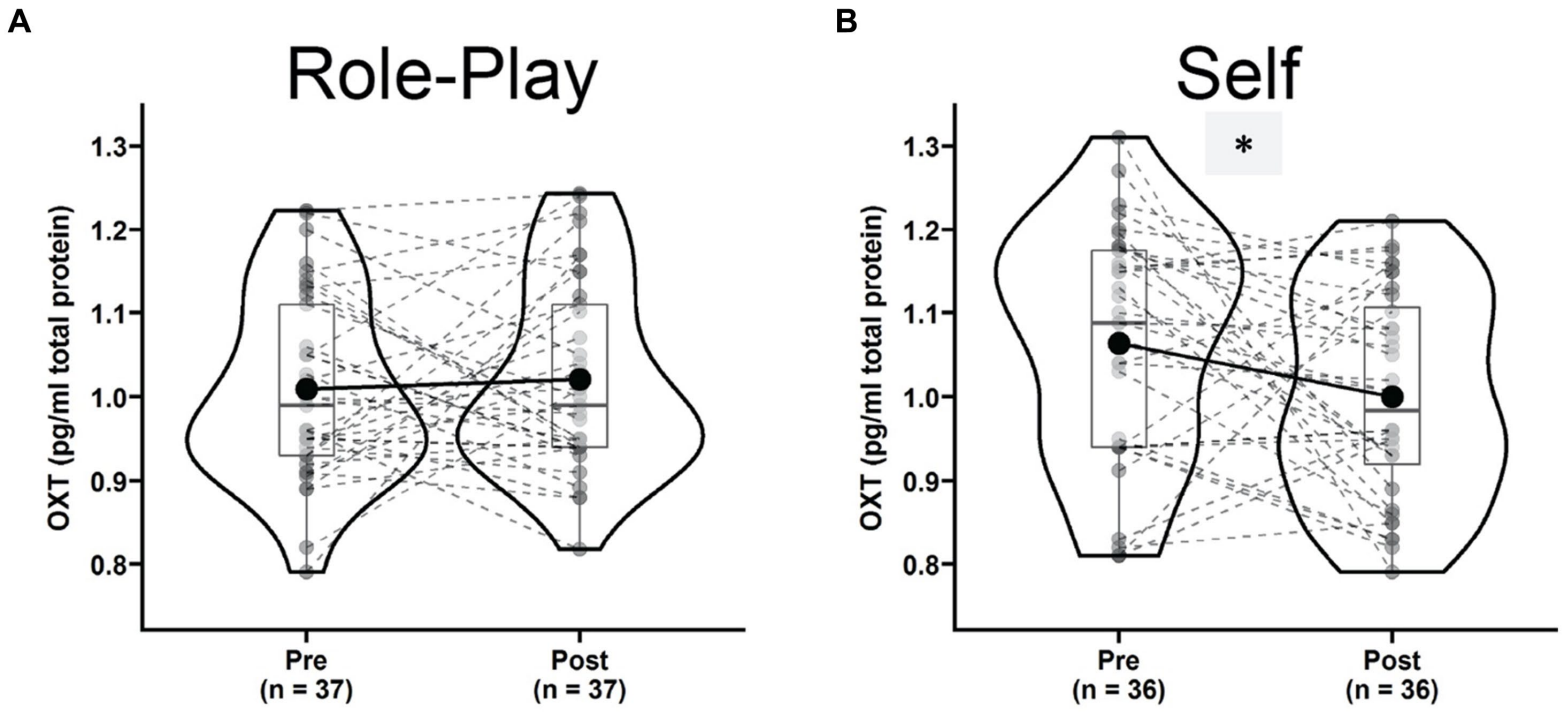
Violin plots for levels of salivary oxytocin dynamic in the role-play condition **(A)** and self condition **(B)** comparing pre (left) versus post (right) states. ^*^Indicates a significant dynamic.

Oxytocin levels presented a small non-significant increase after “Role-play” [Figure 7A, Pre: 1.01 ± 0.11, Post: 1.02 ± 0.11, *t*(36) = 0.50, *p* = 0.623, *g* = 0.08, 95% CI = (0.24, 0.40), power_*α* = 0.05_ = 0.076]. However, the “Self” condition was associated with a significant decrease in salivary oxytocin levels [Figure 7B, Pre: 1.06 ± 0.13, Post: 1.00 ± 0.12, *t*(35) = −2.23, *p* = 0.032, *g* = −0.36, 95% CI = (−0.70, −0.03), power_*α* = 0.05_ = 0.564]. Although the two experimental conditions showed opposite trends, the Time × Condition interaction in repeated measures ANOVA failed to reach significance [*F*(1,33) = 3.472, *p* = 0.071, *η*^2^_g_ = 0.023, power_*α* = 0.05_ = 0.450].

No significant differences were found in pre-intervention between conditions on any of the outcome measures.

### Association between basal salivary oxytocin and the effects of role-play

We also investigated to see if changes in outcome measures could be accounted for by pre-intervention anxiety states and oxytocin levels. Thus, we fitted linear regression models with dynamic (change) scores as a dependent variable and pre-intervention measures as a covariate. Higher anxiety at pre-intervention predicted significantly lower benefits in Positive Affect for “Self” [*β* = −1.16, *t*(33) = −2.44, *p* = 0.024], but not for “Role-play” [*β* = −0.13, *t*(34) = −1.77, *p* = 0.08].

Conversely, higher oxytocin predicted significantly larger gains in Positive Affect for the “Role-play” condition [*β* = 16.22, *t*(33) = 2.52, *p* = 0.017], but not for “Self” [*β* = 7.05, *t*(32) = 1.40, *p* = 0.170].

To examine the indirect effects of time (i.e., pre/post condition) on changes in prosocial attitudes via modifications in positive affect, we applied the recently developed within participant mediation framework developed by Yzerbyt et al. (2018). Inferences about within-participant mediation are based on a series of hypothesis tests (i.e., component approach; Judd et al., 2001) as to not inflate type I errors (Yzerbyt et al., 2018). First, it is required to establish the presence of an indirect effect by means of the significance of both individual components (i.e., the joint-significance test concerning paths *a* and *b*), and only after proceeding with Monte Carlo resampling to compute the confidence interval for the indirect effect (i.e., the product of the two estimated components –*ab*). This analysis was conducted using the *JSmediation* package for R (Yzerbyt et al., 2018).

**Figure 8:**
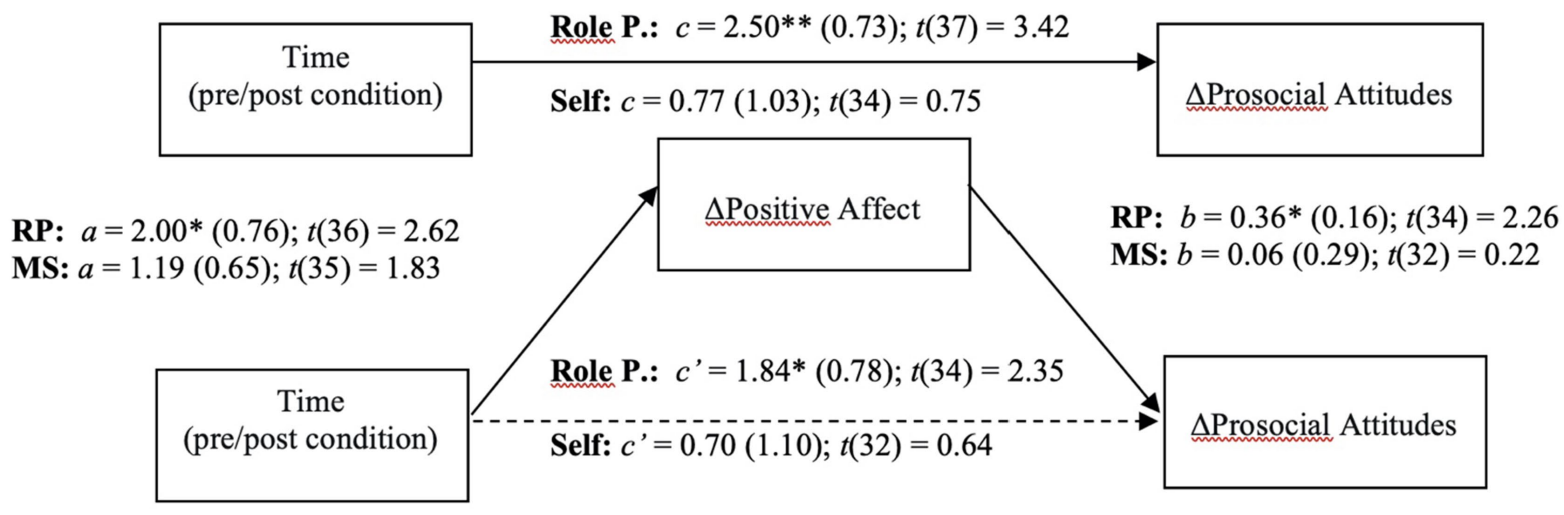
Mediation model of positive affect on prosocial attitudes dynamic.

The applied within-participant mediation model confirmed that observed increases in positive affect accounted for the increases in prosocial attitudes (Figure 8). The within-participant indirect effect was estimated by Monte Carlo method and was found to be significant [*ab* = 0.71; 95% CI = (0.04, 1.69)] only for the “Role-play” condition, while the same effect was non-significant for the “Self” condition.

As expected, prosocial attitudes were also linked to oxytocin levels. Patterns of correlation among all variables are presented in Supplementary material 2. Prosocial attitudes showed a very weak nonsignificant correlation with oxytocin at baseline [“Self”: *r* = −0.06, 95% CI = (−0.38, 0.28); “Role-play”: *r* = −0.03, 95% CI = (−0.35, 0.30)]. This correlation grew consistently after both conditions but was significant only for “Role-play” [*r* = 0.45, 95% CI = (0.15, 0.68), *p* < 0.01], while nonsignificant for “Self” [*r* = 0.24, 95% CI = (−0.11, 0.54), *p* > 0.05]. Differences in correlations between pre and post variables were investigated using *Z* scores and corresponding *p*-values computed with Silver et al. (2004) modification of Dunn and Clark’s (1969) *z* using a back-transformed average Fisher’s (1921) *Z* procedure with, Zou’s (2007) confidence interval showing uncertainty around correlation differences. The increase in the correlation coefficient of prosocial attitudes and oxytocin from pre to post was significant for “Role-play” [*Z* = −2.186, *p* = 0.028, Zou’s CI = (−0.839, −0.066)], but not for “Self” [*Z* = −1.311, *p* = 0.189, Zou’s CI = (−0.701, 0.143)].

## Discussion

In our study, we developed a standardized method to induce role-play in a reproducible and applicable manner. We aimed to investigate the effects of role-play on prosocial attitudes and affect, while also examining the potential modulation by oxytocin, a neurohormone known for its impact on prosocial behavior and anxiety.

To differentiate the effects of role-play from more general forms of pretend play, we created a control task also based on answering questions centered around “dramatic action” comforting the person in front of the participant, subjects in the control condition answering as themselves and in the “Role-play” condition subjects answering from the perspective of the fictional persona. By employing this paradigm, we could discern the specific impacts of pretend play social interaction with and without role-play. Our findings revealed that role-play had a significantly increasing effect on positive affect, dependent on baseline salivary oxytocin levels. This modulatory effect was not observed in the control condition.

Based on CES ratings, we can say that the proposed fictional character was perceived as having a positive affective state (rated with means of 77.1 happy and 80.8 agreeable) and characterized by behaviors that are generally considered prosocial (such as caring, helping, and doing good for the others) with means higher than 80.

### Role-play and emotion regulation

For early developmental psychologists role-play, pretend play, and emotion regulation were ontogenetically intertwined (Piaget, 1952; Vygotsky et al., 1978). Play itself is suggested to catalyze positive emotions (Panksepp, 1993, 2000; Magnuson and Barnett, 2013; Olčar, 2013). Social interaction in multiple forms including synchrony can also produce positive affect (McIntyre et al., 1991; Papasteri et al., 2020) with effects determined by interaction valence, personality traits, and biological factors (McIntyre et al., 1991; Berry and Hansen, 1996; Papasteri et al., 2020; Monninger et al., 2022). In our study, both experimental and control conditions were social and based on pretend play, but the positive emotion increase was significant only for subjects involved in the “Role-play” condition. Negative affect significantly decreased in both conditions while anxiety levels were significantly lower only at the end of the “Role-play” condition. Multiple potential processes, both inhibitory as well as automatic self-soothing, could be consistent with our observations of increased positive and decreased negative affective states after role-play.

The observed decrease in negative affect and anxiety levels at the end of the “Role-play” condition can be attributed to the sense of relief that comes from completing a more challenging task, such as “Role-play” compared to “Self.” Despite the potential perception of a role-playing task as stressful, anxiety levels after the role-play session were significantly lower than at the beginning. Negative emotions are sometimes associated with theatrical performance, and they can be attributed to the cognitive dissonance produced by discrepancies between personal beliefs and performed actions (Haarhoff, 2018), as well as to stress induced by the stakes involved, especially in professional theatre. To minimize potential task perception bias, participants completed questionnaires before listening to the instructions in our experimental design.

More importantly, our linear regression models showed that anxiety levels at the beginning of the “Self” condition predicted lower gains in positive affect, but not for the “Role-play” condition. This suggests that role-play generates a positive affect also for participants experiencing anxiety at the beginning of the task, an aspect not found in the “Self” condition. This points to a possible specificity of affect changes for the “Role-play” interaction.

Our explanation for the significant increase in positive emotions exclusively in the “Role-play” condition, but not in the “Self” condition is mainly to be attributed to the emotional merger between the participant’s affective state and that of the fictional character. If this assumption holds, the impact of role-play on affective experiences would be contingent upon the emotional valence depicted by the character. A previous study also shows that affect outcomes of role-play are dependent on the emotional content of the role-play, theatrical representations of aggressive behavior increasing self-reported levels of depression and hostility both for spectators and performers (Berceanu et al., 2020), information that argues for the idea of the “emotional valence merger.”

Emotion regulation is thought to include the use of both effortful and automatic processes that reduce the intensity or frequency of certain emotional states (e.g., lability) and increase the ability to generate and sustain other emotional states (Hadley et al., 2019). It is suggested that role-play may provide an “unusual opportunity” to learn to maintain “comfortable and stimulating” levels of emotional arousal and that successfully doing so will also lead to rewarding positive emotions (Fein, 1987, 1989).

Role-play involves attention toward the action of the play, “as ifs” as well as other aspects of performance, in our case, *comforting* the experimenter and answering from the fictional character’s perspective and his character. Role-play necessitates a sophisticated and robust level of attention, encompassing attentional orienting, episodic retrieval, and mental imagery. Collectively, these cognitive processes give rise to a proposed form of “split consciousness” experienced by the actor (Kemp, 2012; Brown et al., 2019). This brings another possible explanation for the observed decrease in anxiety levels. The decrease could be attributed to the shift in attention from self during role-play to other tasks needing a strong allocation of attentional resources required by role-play. This attentional shift from self could contribute to a decrease in perceived anxiety.

Role-play is frequently thought to be associated with enhancing the capacity for regulating emotions (Goldstein and Winner, 2012; Goldstein, 2018; Mirodan, 2018). Due to its inherent complexity, the mechanisms through which role-play fosters the development of emotion regulation capacities remain an open question. It is uncertain whether this effect is specifically attributed to the portrayal of a fictional character or if it is related to other factors common to various forms of pretend play. Some researchers propose that actors involved in role-play will consciously down-regulate their own emotions and up-regulate the ones characteristic to the fictional persona (Goldstein, 2009, 2018; McDonald et al., 2020), in the context of our experiment up-regulating their positive affect to be congruent to the positive emotional state they perceived as characterizing the fictional persona.

### Role-play and opinion change

In our study, we observed significant changes in APS scores at the end of the “Role-play” condition, but not in the “Self” condition. We used the APS to observe if attitudes toward prosocial behavior would be changed by role-play in contact with the opinions of the fictional character. Gains in positive affect following role-play were correlated with increases in prosocial attitudes scores.

APS items reflect behaviors and feelings that can be traced to four types of actions: sharing, helping, taking care of, and feeling emphatic. The fictional character in our study would always seek to help others, always consider that others will help them, will always be empathic, willing to share with everybody, and care for both humans and animals. The “Role-play” condition would put participants in the condition of stating that such behaviors, actions, and feelings would be characteristic of them, facilitating opinion change.

Previous studies have shown a contamination effect from the fictional characters on personal attitudes, beliefs, and opinions (Janis and King, 1954; King and Janis, 1956; Janis and Mann, 1965; Ianì, 2019). As expected from our hypothesis, the higher positive affect was associated with higher changes in prosocial behavior as measured by APS, confirming the facilitation of opinion changes through role-play experiences, as well as the importance of positive affect in the process.

Cognitive schemas, normative beliefs, world schemas, and scripts are stored in memory and activated by salient cues and further act as guides for social behavior (Huesmann, 1998). Cognitive scripts incorporate declarative and procedural knowledge and prescribing responses. As the activation of scripts repeatedly supports responses that have consistent and desirable consequences, the scripts themselves become more salient and readily accessible (Huesmann, 1998). The more extensive and primed the networks are, the more accessible they get (Huesmann, 1998). The scripts and schemas are shaped and reinforced into one’s long-term memory by enactive learning (Huesmann, 1998), so in a role-play action one’s cognitive schemas might be re-shaped by cognitive schemas proposed by the induction script and enacted through verbal action.

Even if subjects remain seated, choosing a hat and wearing it as an object characteristic for the character could provide a “physical interface” with the fictional character. Working with real objects is considered key in obtaining fragmentary “real” embodied reactions to the “as if” proposed by the situation and the character (Stanislavski, 2010). Further studies could also focus on clarifying if using objects and costumes in role-play increases merger.

In sum, our findings regarding role-play outcomes on emotions and prosocial attitudes point to a “merger” effect between the fictional character and the self of the subjects at cognitive and affective levels. Since our participants did not have prior training in acting, they could be less prepared to block this type of merger by quarantine. The modulator effect of positive emotions for changes in prosocial attitudes is in line with the proposal that a positive affective environment strongly contributes to learning and cognitive change and is proposed as a factor of change in psychotherapy (Fitzpatrick and Stalikas, 2008), with recent empirical proof (McNeil and Repetti, 2022).

### Oxytocin modulation of affect

Oxytocin was linked to positive emotions in animals (Uvnäs-Moberg, 1998) and in humans (Alexander et al., 2021). Other studies found that higher levels of oxytocin monitored in saliva predicted higher positive emotions after an acute stress induction protocol (Young Kuchenbecker et al., 2021), and positive emotions induced by storytelling correlated with higher levels of salivary oxytocin in hospitalized children (Brockington et al., 2021).

Our data showed that higher levels of oxytocin predicted larger gains in affect at the end of the “Role-play” condition but not for the “Self” condition. Several mechanisms in which oxytocin was previously shown to be involved could explain this association, linking social interaction to reward and to prosocial behavior (Bartz et al., 2011; Shamay-Tsoory and Abu-Akel, 2016). One potential explanation is that individuals with higher oxytocin levels are more sociable and readily engage in the role-play task as it was proposed that higher levels of oxytocin would be correlated with higher levels of prosocial behavior (Bartz et al., 2011; Marsh et al., 2021). However, we did not observe such a direct relationship but a dynamic one, pointing to a more complex model, also coherent with a complex, non-mechanistic role for oxytocin involvement in behavior.

A well-known neural substrate for positive affect is the mesolimbic reward system, specifically the release of dopamine from ventral tegmental area (VTA) into the nucleus accumbens (Arias-Carrión et al., 2010). Work in animal models has shown that both structures express receptors for the oxytocin hormone and that the activity of oxytocin fibers can trigger the release of dopamine in this mesolimbic circuit (Dölen et al., 2013; Hung et al., 2017). Modulation of VTA activity by oxytocin was also observed in humans in a task involving rewarding and non-rewarding social stimuli (Groppe et al., 2013). Considering role-play as a form of play, then reward anticipation, specific to play, might be involved in some of the observed outcomes. Further studies are needed to elucidate the possible impact of interactions of play, reward, social interaction, role-play circuitry, and their neurohormonal substrate brought to attention by the observed moderating effects of oxytocin in our role-play condition for the gains in positive affect.

Although we did not observe a significant effect of role-play on salivary oxytocin levels, trends post “Role-play” and post-control appear to be opposed, with a slight increase in the “Role-play” and a significant decrease for the “Self” condition. A possible increase connected with a subgroup of subjects in the “Role-play” might be concealed by the task or individual parameters that drive a decrease in oxytocin levels for some of the subjects. However, the higher levels of oxytocin before the experiment predicted higher gains in positive affect for post “Role-play” but not for the control condition, indicating a specific oxytocinergic substrate involved in the role-play.

Evaluating the arguments for different explanations for the observed changes at the affective and cognitive levels, the most compelling is that of a “merger effect” between the self of the subject and the fictional persona. Positive affect associated with the “Role-play” condition facilitates stronger gains for subjects with higher levels of oxytocin. “Role-play” provides a positive context to the social interaction. Context was previously reported as important for behavior outcomes of social interactions modulated oxytocin with both prosocial and antisocial outcomes reported (Bartz et al., 2011), dependent on the intensity of the stimuli (Uvnäs-Moberg et al., 2014), emotional context (Baettig et al., 2020), gender and previous social relationship (Handlin et al., 2023).

To our knowledge, this is the first study trying to observe the oxytocin substrate in role-play. We observed a complex model linking oxytocin levels and positive affect, which was further linked to increases in prosocial attitudes. Further studies are needed to gain more knowledge of neural and psychological processes involved in Role-play, combining them with neuro-imaging and neurochemical component analyses. Such studies would bring light to crucial aspects of role-play contributing to a more impactful use of Role-play in psychotherapy using or impacting the neurohormonal substrate.

In acting, as well as in other forms of role-play, for instance, in RPGs (role-play games) or LARP (live-faction role-play), different strategies can be used to induce role-play (Kipper and Har-Even, 1984; Kipper, 1986). Approaches and strategies in role-play can be very different, as in mime, realist acting, or shamanic rituals, and each of these approaches of role-play could involve a specific psychological process and subsequently different brain processing substrates. Despite such a diverse range of creative processes for portraying others in action, it is proposed that they all share a common ground at the level of “proto-acting” (Brown, 2017). Understanding the common processes requires a holistic approach, interconnecting psychological, functional, and biochemical aspects.

### Limitations

With a small sample size (*n* = 41), our study showed significant results at the level of affect and prosocial attitudes and a mediation effect of oxytocin for positive affect gains for subjects when in the role-play condition. To confirm observed effects, it would be important to replicate this study on a larger sample size, including in the design of a control task not involving pretend play. It would be very important to evaluate the level of adherence and accomplishment of the proposed “as if” instructions. Although the merger effect could explain our findings, it is hard to conclude that the observed effect is truly a “merger” effect, or if the result of enjoyment is brought by role-play since we did not control either how immersed subjects were in the task or the level at which they accomplished instructions in each condition. Other aspects of social interaction such as emotional contagion or synchrony could be involved in observed changes but as our experiment did not control for them, future studies will be needed to further clarify the mechanisms involved.

Our study did not control for participant gender impact on psychological and biochemical measures. Similarly, we did not control for the gender of the interviewer and experimenter—all interviewers in our study were women. A further study accounting for gender influence is of interest and it should include both gender experimenters.

Participants in our study did not have prior theatrical training so the novelty of the task, including using props, like the funny hat, might have an effect at affect levels that may not transfer to other populations, such as professional actors. This also brought on another limitation: the lack of experience of subjects in role-play increased the chance that our results are specific to the group and could not be replicated in a population with strong acting experience. Professional actors are supposed to have a stronger ability to set boundaries between self and fictional character would prevent contamination effects between the two, both at affect and cognitive levels. Since this is a question of high importance for performers and teaching strategies in theatre it is an aspect that needs to be further investigated.

The questionnaire used to measure prosocial attitudes (APS) was not designed for repeated measures, however, in our study, it was used with a different purpose than its validation bringing a limitation to our data. We consider the measure as a tool to observe opinion change, previously reported to be produced by role-play (Janis and King, 1954; Janis and Mann, 1965). Our choice was to use this tool since we hypothesized that role-play will impact attitudes.

A larger sample size could bring more information on the role of oxytocin, where we observe opposed dynamics between “Self” and “Role-play” conditions. Some trends can be observed in the pre-post evolution of oxytocin, significantly lower at the end of the “Self” condition, when compared to levels observed at the beginning, and higher at the end of the “Role-play” condition, compared to levels at the beginning, but not reaching significance levels.

Monitoring oxytocin levels in saliva has a lot of advantages, especially in experiments targeting stress or anxiety levels. The collection of blood plasma or urine is more stressful for subjects. Measured levels of oxytocin in saliva are correlated with behavioral outcomes of human interactions and activities such as physical exercise, sexual stimulation, induced social stress, touching, mother-infant, and father-infant interaction, etc., correlating also with behavioral aspects (Jurek and Neumann, 2018). Moreover, while salivary oxytocin does not perfectly reflect central oxytocin levels, the levels of oxytocin in saliva show a stronger correlation with brain oxytocin (rho = 0.6, *p* < 0.001) than plasma oxytocin levels (rho = 0.1, *p* = 0.003) (Martin et al., 2018). This indicates that changes in brain oxytocin levels would be reflected by changes in salivary oxytocin.

In our study, we chose oxytocin as a biochemical measurement due to its involvement in social interaction, prosocial behavior, and affective states in our key targets, however, other neurotransmitters such as orexin, dopamine, serotonin and are presumed to have an important role. In the case of prosocial attitudes, both oxytocin and serotonin action in the nucleus accumbens have been implicated in driving increased social engagement (Walsh et al., 2018; Froemke and Young, 2021). In humans, changes in central neurotransmitters are difficult to investigate. However, future work could determine if medically increasing brain serotonin (for example, by MDMA administration) could saturate the effects of role-play on prosocial attitudes. Investigating functional brain aspects of the role-play psychological process as well as investigating the role of reward, flow, and the neurochemical substrate would bring a holistic approach to role-play important for understanding this complex creative process used in art, therapy, and daily life.

## Data availability statement

The raw data supporting the conclusions of this article will be made available by the authors, without undue reservation.

## Ethics statement

The studies involving humans were approved by National University for Theatre and Film Ion Luca Caragiale Bucharest Ethics Committee. The studies were conducted in accordance with the local legislation and institutional requirements. The participants provided their written informed consent to participate in this study. Written informed consent was obtained from the individual(s) for the publication of any potentially identifiable images or data included in this article.

## Author contributions

AB: Conceptualization, Methodology, Writing – original draft, Project administration, Investigation, Funding acquisition. CPa: Writing – review & editing, Writing – original draft, Formal analysis. AS: Writing – review & editing, Investigation. RB: Writing – review & editing, Investigation. DN: Writing – review & editing, Investigation. CPo: Writing – review & editing, Formal analysis. IC: Conceptualization, Writing – review & editing, Supervision, Investigation, Funding acquisition. RF: Conceptualization, Writing – review & editing, Supervision, Funding acquisition.
