# Supplementary material for: Oxytocin predicts positive affect gains in a role-play interaction
Source: Front Psychol. 2024 May 30;15:1258254. doi: 10.3389/fpsyg.2024.1258254 (PMC11169887; doi:10.3389/fpsyg.2024.1258254)
Supplement: Supplementary file 1 [file Table_1.docx]

Supplementary Material 1

Oxytocin moderates positive affect gains in a role-play interaction

**Alexandru I. Berceanu*^1,2^, Claudiu Papasteri^1,3^, Alexandra Sofonea^1^, Romina Boldasu^4^, Diana Nita^1^, Cătălina Poalelungi^1,5^, Robert Froemke^1,6^, Ioana Carcea^1,7^**

^1^ Cognitive Development and Applied Psychology through Immersive Experiences, LDCAPEI,

CINETic Centre, National University of Theatre and Film IL Caragiale, Bucharest, Romania,

^2^ Department for Animation and Interactivity, National University of Theatre and Film IL Caragiale, Bucharest, Romania,

^3^ Department of Psychology, Faculty of Psychology and Educational Sciences, University of Bucharest, Bucharest, Romania National Institute of Endocrinology C. I. Parhon​​, Bucharest, Romania

^4^ Acting Department Theatre Faculty, National University of Theatre and Film IL Caragiale, Bucharest, Romania

^5^ National Institute of Endocrinology C. I. Parhon​​, Bucharest, Romania

Department for Animation and Interactivity, National University of Theatre and Film IL Caragiale, Bucharest, Romania

^6^ Skirball Institute for Biomolecular, School of Medicine, New York University

^7^ Brain Health Institute, Department of Pharmacology, Physiology and Neuroscience, New Jersey Medical School, Rutgers, The State University of New Jersey, Newark, NJ, United States

*** Correspondence:
Claudiu Papasteri^1,3^**
claudiu.papasteri@gmail.com;

Berceanu I. Alexandru berceanu.cinetic@unatc.ro

Keywords: *role-play, oxytocin, positive affect, prosocial attitudes, emotion regulation*.

# Supplementary Materials 1

This is the complete set of instructions that were played to participants on each condition.

**Day one:** In the training session, all instructions were listened together with the trainer, and the participant with the participant asked questions if they considered them necessary.

**Day two (If assigned “Self” condition):** General Instruction+ Dramatic action condition.

**Day three (If assigned “Role-play” condition):** General Instruction+Role-play instruction+Fictional Persona Story+Dramatic Action.

**General instruction:** The current experiment does not test your artistic abilities. It will put you in a common life situation where you know how to react without the need of any particular ability. Try to behave logically and consistently with the instructions. If at any point during the instructions you have uncertainties, please request more information from the experiment administrator. It is important after start to proceed smoothly without interrupting or asking for information from your partner.

**“Role-play” instruction:** I will describe a character to you. At the end of the story, you will put on his hat, which is on the table, and you will respond at all times as if you were him/her. Finally, you will take off the hat and become yourself again. While wearing the hat, try to view the world and everything that happens from his perspective and act and answer as he/she would to all questions. He/she will have your voice and your body; there is no need to make any extra effort other than wearing his hat.

**“Self”** **instruction:** A person will enter the room. She will ask you some questions. Answer from your perspective”.

**Dramatic action instruction (DA):** "The person entering the room is scheduled for a surgical procedure shortly after the interview, to comfort her with your answers."

### Unlucky - Role-play induction script

The main elements of the story were inspired by different clown characters and typologies like Pierrot, Chaplin’s 'The Tramp', and Chekov’s Epihodov.

*People say he's the unluckiest, but he thinks he's the luckiest. He was always followed by bad luck, but he never cared about it. I think every time he drinks a cup of water, he finds a fly in it but he never gets upset. He just jokes and says that one day he will catch the one who puts it there. If he wants to use a fork he will surely break it. That's why he never gets his hands on a hammer or an ax.*

*Once he was at a restaurant, he had just gotten a straw hat and he was very happy about it. In his soup, he noticed a large hair. He points to the waiter who is worried about the situation. The unlucky one says, "Leave it, there's no upset, I can handle it." The waiter, "No, it is not possible. Please". The waiter takes the plate and withdraws. Physics also has its laws, there was no chance that the soup would reach a man's head, but just then the next-door lady`s dog barked, the waiter got scared and the soup landed directly on his new straw hat. The unlucky man was not upset, the truth is that now he eats there for free every time. The lady with the puppy was the owner of the place.*

*This hat still had many adventures. In fact, all his hats had many adventures, another in his place would give up wearing but he never takes it off his head. He says it's part of his personality. That makes him funny.*

*I asked him when he started to get unlucky and he told me some childhood memories. His first memory was with a leather ball while playing football with his friends. I don't know how a ball could be big enough to overturn it and tear down the whole net from goal to goal. However, the children loved him even then, even if they had to make a new football goal. At school, when the teacher first took him to the blackboard, he sneezed so hard that he hit his head on the blackboard. Imagine how much everyone in the class laughed. He was not upset, moreover, he turned it into a joke that he made every break until everyone burst out laughing.*

*His favorite joke is that everyone loves him but no one lends him anything if they know him. I lend him anything, maybe sometimes or every time, they broke down, but I never felt damaged. After meeting him, I feel happy. I don't know if he has a family, but someone is always with him and he feels good. He used to have a puppy. That clown big dog! He was curly, he followed him everywhere and he looked as if he winked at you all the time.*

*Sometimes I thought he was a superficial guy, but he explained where his joy came from. He feels that there is a lot of security in his life. He knows exactly what is going to happen because he says he sees bad luck long before he comes. He feels at peace with his life and enjoys everything. It's true, he never flies because he loves others too much. He can be sure of a few things: he will never be trampled by the train because it will break down before it reaches him, but also that if he wins the lottery, he will lose the ticket.*

*I don't think he'll ever be alone, no one will get mad at him, or he'll be forgiven and he'll never be sad. Any disaster, no matter how big, no matter how many things collapse around him, no matter how unlucky he is, the fun is maximum and, in the end, he stands. Every time I am sad, I would like to be by his side, I know that I will see things better. When you know that someone is so unlucky but happy, it helps. He and his straw hat.*

### Training Questions:

1. What is your favorite pet?
2. How do you choose to spend your birthday?
3. How are you doing with the money?
4. Tell me a funny memory from your life.
5. What do people say about you?
6. What do you want most?
7. If you had a magical power that you would want?
8. What makes you the angriest?
9. How do you relax?
10. I forgot the light on, what do we do?
11. You are in a dark room, there are noises from behind a door. What are you doing?
12. My mother is very angry. What should I do?
13. I missed the last train. What to do?

**These questions were used in the training session by the experimenter to familiarize the subjects with the tasks. Six of them would be in the ‘self ‘condition and six in the role-play condition.**

### Interview Questions

1. What do you like to wear?

2. How do you make a living?

3. What do you have in your pockets?

4. When you open the refrigerator door, what do you see?

5. When you open the window of your home, what do you see?

6. What is your preferred means of transportation?

7. Tell me something about your pet.

8. Tell me a memory with a friend.

9. Tell me a very pleasant memory from your life.

10. Tell me a story that scared you.

11. Tell me a bizarre memory from your life.

12. What is your highest quality?

13. What is your biggest flaw?

14. What do you think about yourself?

15. What do your friends think of you?

16. What color do you like the most?

17. What is your favorite song?

18. Where would you most like to go?

19. What cheers you up the most?

20. Why are you most afraid of?

21. What reassures you?

22. What is your dream?

23. In front of you is a giant window. What do you do?

24. Someone's wallet falls next to you. What do you do?

25. There is a kitten in the rain in the street. What do you do??

26. Thou didst wander in the way in the forest, in the distance a light was seen, and a dog barked. What do you do?

27. A child is playing on the edge of a lake with the ball. What are you doing?

28. I lost my house keys. What should we do?

29. I forgot the pot on the fire. What should we do?

30. I lost the documents. What to do?

**These questions were used both in the role-play condition and in the self condition.**

**
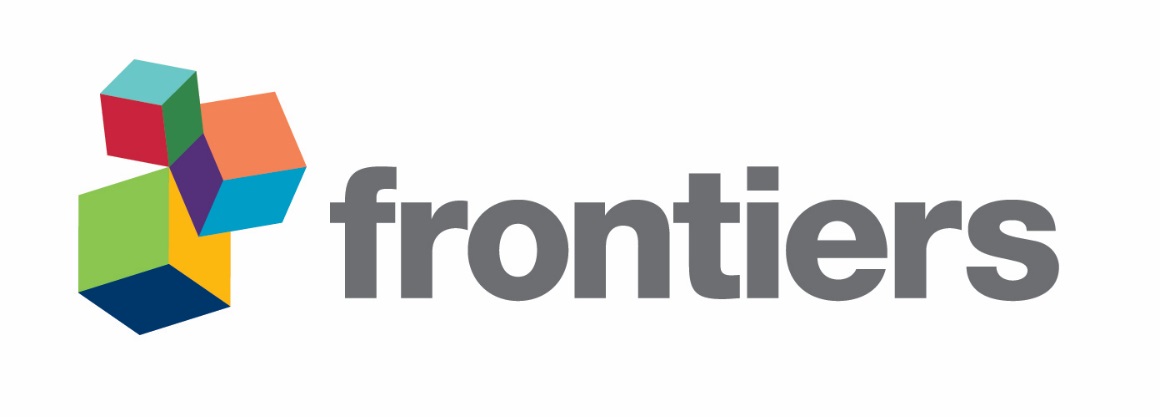
**
