# Supplementary material for: Oxytocin predicts positive affect gains in a role-play interaction
Source: Front Psychol. 2024 May 30;15:1258254. doi: 10.3389/fpsyg.2024.1258254 (PMC11169887; doi:10.3389/fpsyg.2024.1258254)
Supplement: Supplementary file 2 [file Table_2.docx]

Supplementary Material 2

Oxytocin moderates positive affect gains in a role-play interaction

**Alexandru I. Berceanu*^1,5^, Claudiu Papasteri^1,2^, Alexandra Sofonea^1^, Romina Boldasu^6^, Diana Nita^1^, Cătălina Poalelungi^1,3^, Robert Froemke^1,7^, Ioana Carcea^1,4^**

^1^ Cognitive Development and Applied Psychology through Immersive Experiences, LDCAPEI,

CINETic Centre, National University of Theatre and Film IL Caragiale, Bucharest, Romania,

^2^ Department of Psychology, Faculty of Psychology and Educational Sciences, University of Bucharest, Bucharest, Romania,

^3^ National Institute of Endocrinology C. I. Parhon​​, Bucharest, Romania

^4^ Brain Health Institute, Department of Pharmacology, Physiology and Neuroscience, New Jersey Medical School, Rutgers, The State University of New Jersey, Newark, NJ, United States

^5^ Department for Animation and Interactivity, National University of Theatre and Film IL Caragiale, Bucharest, Romania

^6^ Acting Department Theatre Faculty, National University of Theatre and Film IL Caragiale, Bucharest, Romania

^7^ Skirball Institute for Biomolecular, School of Medicine, New York University

*Table 1. ‘Self’ condition: correlations with confidence intervals of pre-test and post-test measures*

| Variables | 1 | 2 | 3 | 4 | 5 | 6 | 7 | 8 | 9 |
| --- | --- | --- | --- | --- | --- | --- | --- | --- | --- |
|  |  |  |  |  |  |  |  |  |  |
| 1. PA Pre |  |  |  |  |  |  |  |  |  |
|  |  |  |  |  |  |  |  |  |  |
| 2. NA Pre | -.03 |  |  |  |  |  |  |  |  |
|  | [-.35, .30] |  |  |  |  |  |  |  |  |
|  |  |  |  |  |  |  |  |  |  |
| 3. PSA Pre | .30 | -.06 |  |  |  |  |  |  |  |
|  | [-.03, .57] | [-.38, .27] |  |  |  |  |  |  |  |
|  |  |  |  |  |  |  |  |  |  |
| 4. ANX Pre | -.40* | .58** | -.16 |  |  |  |  |  |  |
|  | [-.64, -.09] | [.32, .76] | [-.46, .18] |  |  |  |  |  |  |
|  |  |  |  |  |  |  |  |  |  |
| 5. OXT Pre | .06 | .20 | -.06 | .01 |  |  |  |  |  |
|  | [-.27, .38] | [-.13, .50] | [-.38, .28] | [-.32, .34] |  |  |  |  |  |
|  |  |  |  |  |  |  |  |  |  |
| 6. PA Post | .78** | -.12 | .29 | -.54** | -.04 |  |  |  |  |
|  | [.60, .88] | [-.43, .22] | [-.04, .57] | [-.74, -.25] | [-.37, .30] |  |  |  |  |
|  |  |  |  |  |  |  |  |  |  |
| 7. NA Post | .00 | .77** | -.18 | .53** | .32 | -.15 |  |  |  |
|  | [-.32, .33] | [.60, .88] | [-.48, .16] | [.24, .73] | [-.02, .59] | [-.46, .19] |  |  |  |
|  |  |  |  |  |  |  |  |  |  |
| 8. PSA Post | .27 | .10 | .87** | -.12 | -.00 | .29 | -.06 |  |  |
|  | [-.07, .55] | [-.24, .42] | [.76, .93] | [-.43, .22] | [-.34, .34] | [-.05, .57] | [-.39, .28] |  |  |
|  |  |  |  |  |  |  |  |  |  |
| 9. ANX Post | -.19 | .45* | -.39* | .64** | .08 | -.46* | .77** | -.33 |  |
|  | [-.54, .21] | [.07, .71] | [-.68, -.00] | [.34, .82] | [-.33, .46] | [-.72, -.09] | [.54, .89] | [-.64, .07] |  |
|  |  |  |  |  |  |  |  |  |  |
| 10. OXT Post | -.11 | -.03 | .17 | -.07 | .14 | .05 | -.05 | .24 | -.05 |
|  | [-.42, .23] | [-.36, .30] | [-.16, .48] | [-.39, .26] | [-.20, .45] | [-.29, .38] | [-.38, .29] | [-.11, .54] | [-.44, .35] |
|  |  |  |  |  |  |  |  |  |  |

PA = Positive Affect; NA = Negative Affect; PSA = Prosocial Attitudes; ANX = Anxiety; OXT = Oxytocin.

*Note.* Values in square brackets indicate the 95% confidence interval for each correlation. The confidence interval is a plausible range of population correlations that could have caused the sample correlation (Cumming, 2014). * indicates *p* < .05. ** indicates *p* < .01.

*Table 2. ‘Role-play’ condition: correlations with confidence intervals of pre-test and post-test measures*

| Variables | 1 | 2 | 3 | 4 | 5 | 6 | 7 | 8 | 9 |
| --- | --- | --- | --- | --- | --- | --- | --- | --- | --- |
|  |  |  |  |  |  |  |  |  |  |
| 1. PA Pre |  |  |  |  |  |  |  |  |  |
|  |  |  |  |  |  |  |  |  |  |
| 2. NA Pre | -.05 |  |  |  |  |  |  |  |  |
|  | [-.36, .28] |  |  |  |  |  |  |  |  |
|  |  |  |  |  |  |  |  |  |  |
| 3. PSA Pre | .07 | -.29 |  |  |  |  |  |  |  |
|  | [-.25, .38] | [-.56, .03] |  |  |  |  |  |  |  |
|  |  |  |  |  |  |  |  |  |  |
| 4. ANX Pre | -.28 | .74** | -.25 |  |  |  |  |  |  |
|  | [-.55, .04] | [.54, .85] | [-.53, .07] |  |  |  |  |  |  |
|  |  |  |  |  |  |  |  |  |  |
| 5. OXT Pre | -.11 | -.09 | -.03 | -.09 |  |  |  |  |  |
|  | [-.42, .22] | [-.40, .24] | [-.35, .30] | [-.40, .25] |  |  |  |  |  |
|  |  |  |  |  |  |  |  |  |  |
| 6. PA Post | .77** | -.33* | .21 | -.39* | .07 |  |  |  |  |
|  | [.60, .88] | [-.59, -.01] | [-.12, .50] | [-.63, -.08] | [-.27, .39] |  |  |  |  |
|  |  |  |  |  |  |  |  |  |  |
| 7. NA Post | .06 | .88** | -.22 | .68** | -.10 | -.21 |  |  |  |
|  | [-.27, .37] | [.78, .94] | [-.51, .11] | [.46, .82] | [-.41, .24] | [-.50, .13] |  |  |  |
|  |  |  |  |  |  |  |  |  |  |
| 8. PSA Post | .01 | -.33* | .93** | -.27 | -.03 | .22 | -.27 |  |  |
|  | [-.31, .33] | [-.58, -.01] | [.88, .97] | [-.54, .05] | [-.35, .30] | [-.11, .51] | [-.55, .06] |  |  |
|  |  |  |  |  |  |  |  |  |  |
| 9. ANX Post | -.52** | .67** | -.22 | .92** | -.00 | -.65** | .68** | -.26 |  |
|  | [-.74, -.19] | [.41, .83] | [-.54, .16] | [.83, .96] | [-.37, .37] | [-.83, -.37] | [.41, .84] | [-.57, .12] |  |
|  |  |  |  |  |  |  |  |  |  |
| 10. OXT Post | -.08 | -.24 | .43** | -.09 | .16 | .15 | -.17 | .45** | -.16 |
|  | [-.39, .25] | [-.52, .09] | [.13, .66] | [-.40, .24] | [-.17, .46] | [-.18, .46] | [-.47, .17] | [.15, .68] | [-.50, .23] |
|  |  |  |  |  |  |  |  |  |  |

PA = Positive Affect; NA = Negative Affect; PSA = Prosocial Attitudes; ANX = Anxiety; OXT = Oxytocin.

*Note.* Values in square brackets indicate the 95% confidence interval for each correlation. The confidence interval is a plausible range of population correlations that could have caused the sample correlation (Cumming, 2014). * indicates *p* < .05. ** indicates *p* < .01.

*Table 2. ‘Self’ condition: Means (Standard Deviation) of outcome measures*

|  | Pre | | Post | |
| --- | --- | --- | --- | --- |
| Variables | Range | M (SD) | Range | M (SD) |
| Positive Affect | 20-44 | 32.35(4.85) | 23-50 | 33.50(6.19) |
| Negative Affect | 10-29 | 13.91(5.03) | 9-26 | 12.33(3.95) |
| Prosocial Attitudes | 34-78 | 61.64(9.53) | 23-78 | 62.31(12.27) |
| Anxiety | 20-64 | 33.00(10.28) | 20-59 | 30.00(8.78) |
| Oxytocin | 0.81-1.31 | 1.06(0.13) | 0.79-1.21 | 1.00(0.12) |

*Table 3. ‘Role-play’ condition: Means (Standard Deviation) of outcome measures*

|  | Pre | | Post | |
| --- | --- | --- | --- | --- |
| Variables | Range | M (SD) | Range | M (SD) |
| Positive Affect | 19-47 | 31.34(6.47) | 20-50 | 33.67(7.23) |
| Negative Affect | 10-27 | 14.60(4.92) | 10-25 | 13.02(4.33) |
| Prosocial Attitudes | 27-75 | 58.68(12.39) | 28-79 | 61.18(12.36) |
| Anxiety | 20-60 | 34.00(10.54) | 20-53 | 31.24(9.10) |
| Oxytocin | 0.79-1.22 | 1.01(0.11) | 0.82-1.24 | 1.02(0.11) |

*Note:* Mean (Standard Deviation)

*Table 4.* *‘Self’ condition: Pre-test and post-test comparisons of correlations between oxytocin and each of the psychological outcome measures*

| Variables | Pre | Post | Z | P-value | Zou's CI |
| --- | --- | --- | --- | --- | --- |
| Positive Affect | 0.06  [-0.27, 0.38] | 0.05  [-0.29, 0.38] | 0.027 | 0.978 | [-0.428, 0.439] |
| Negative Affect | 0.20  [-0.13, 0.50] | -0.05  [-0.38, 0.29] | 1.086 | 0.277 | [-0.194, 0.666] |
| Prosocial Attitudes | -0.06  [-0.38, 0.28] | 0.24  [-0.11, 0.54] | -1.311 | 0.189 | [-0.701, 0.143] |
| Anxiety | 0.01  [-0.32, 0.34] | -0.05  [-0.44, 0.35] | 0.265 | 0.791 | [-0.385, 0.501] |

*Note.* Values in square brackets indicate the 95% confidence interval for each Pearson correlation coefficient. The confidence interval is a plausible range of population correlations that could have caused the sample correlation (Cumming, 2014). Z scores and corresponding p-values are computed with Silver, Hittner, and May’s (2004) modification of Dunn and Clark’s (1969) z using a backtransformed average Fisher’s (1921) Z procedure. Zou’s (2007) confidence interval shows uncertainty around correlation differences.

*Table 5. ‘Role-play’ condition: Pre-test and post-test comparisons of correlations between oxytocin and each of the psychological outcome measures*

| Variables | Pre | Post | Z | P-value | Zou's CI |
| --- | --- | --- | --- | --- | --- |
| Positive Affect | -0.11  [-0.42, 0.22] | 0.15  [-0.18, 0.46] | -1.177 | 0.239 | [-0.675, 0.178] |
| Negative Affect | -0.09  [-0.40, 0.24] | -0.17  [-0.47, 0.17] | 0.386 | 0.699 | [-0.339, 0.500] |
| Prosocial Attitudes | -0.03  [-0.35, 0.30] | 0.45*  [ 0.15, 0.68] | -2.186 | **0.028** | [-0.839, -0.066] |
| Anxiety | -0.09  [-0.40, 0.25] | -0.16  [-0.50, 0.23] | 0.316 | 0.752 | [-0.352, 0.483] |

*Note.* Values in square brackets indicate the 95% confidence interval for each Pearson correlation coefficient. The confidence interval is a plausible range of population correlations that could have caused the sample correlation (Cumming, 2014). Z scores and corresponding p-values are computed with Silver, Hittner, and May’s (2004) modification of Dunn and Clark’s (1969) z using a backtransformed average Fisher’s (1921) Z procedure. Zou’s (2007) confidence interval shows uncertainty around correlation differences.

* indicates *p* < .05. ** indicates *p* < .01.

References

Cumming, G. (2014). The new statistics: Why and how. *Psychological science*, *25*(1), 7-29. <https://doi.org/10.1177/0956797613504966>

Silver, N. C., Hittner, J. B., & May, K. (2004). Testing dependent correlations with nonoverlapping variables: A Monte Carlo simulation. *Journal of Experimental Education*, *73*, 53-69. <https://doi.org/10.3200/JEXE.71.1.53-70>

Zou, G. Y. (2007). Toward using confidence intervals to compare correlations. *Psychological Methods*, *12*, 399-413. <https://doi.org/10.1037/1082-989X.12.4.399>
